# *Rhizobium etli* Produces Nitrous Oxide by Coupling the Assimilatory and Denitrification Pathways

**DOI:** 10.3389/fmicb.2019.00980

**Published:** 2019-05-07

**Authors:** Alba Hidalgo-García, María J. Torres, Ana Salas, Eulogio J. Bedmar, Lourdes Girard, María J. Delgado

**Affiliations:** ^1^Estación Experimental del Zaidín, Consejo Superior de Investigaciones Científicas, Granada, Spain; ^2^Centro de Ciencias Genómicas, Universidad Nacional Autónoma de México, Cuernavaca, Mexico

**Keywords:** assimilatory nitrate reductase, denitrification, gene expression, soil bacteria, nitrous oxide

## Abstract

More than two-thirds of the powerful greenhouse gas nitrous oxide (N_2_O) emissions from soils can be attributed to microbial denitrification and nitrification processes. Bacterial denitrification reactions are catalyzed by the periplasmic (Nap) or membrane-bound (Nar) nitrate reductases, nitrite reductases (NirK/*cd*_1_Nir), nitric oxide reductases (cNor, qNor/ Cu_A_Nor), and nitrous oxide reductase (Nos) encoded by *nap*/*nar*, *nir*, *nor* and *nos* genes, respectively. *Rhizobium etli* CFN42, the microsymbiont of common bean, is unable to respire nitrate under anoxic conditions and to perform a complete denitrification pathway. This bacterium lacks the *nap*, *nar* and *nos* genes but contains genes encoding NirK and cNor. In this work, we demonstrated that *R. etli* is able to grow with nitrate as the sole nitrogen source under aerobic and microoxic conditions. Genetic and functional characterization of a gene located in the *R. etli* chromosome and annotated as *narB* demonstrated that growth under aerobic or microoxic conditions with nitrate as nitrogen source as well as nitrate reductase activity requires NarB. In addition to be involved in nitrate assimilation, NarB is also required for NO and N_2_O production by NirK and cNor, respectively, in cells grown microoxically with nitrate as the only N source. Furthermore, β-glucuronidase activity from *nirK::uidA* and *norC::uidA* fusions, as well as NorC expression and Nir and Nor activities revealed that expression of *nor* genes under microoxic conditions also depends on nitrate reduction by NarB. Our results suggest that nitrite produced by NarB from assimilatory nitrate reduction is detoxified by NirK and cNor denitrifying enzymes that convert nitrite into NO which in turn is reduced to N_2_O, respectively.

## Introduction

Nitrous oxide (N_2_O) is a powerful greenhouse gas (GHG) and a major cause of ozone layer depletion (Ravishankara et al., 2009) with an atmospheric lifetime of 114 years and an estimated 300-fold greater potential for global warming compared with that of carbon dioxide (CO_2_), based on its radiative capacity (Intergovernmental Panel on Climate Change [IPCC], 2014). Human activities such as agriculture, fossil fuel combustion, wastewater management and industrial processes have provoked escalating emissions of N_2_O which contribute to climate change. More than 60% of N_2_O emissions globally are emitted from agricultural soils (Smith et al., 2012). This contribution has been amplified through the so-called “green revolution,” which has increased the presence of nitrogen (N) in soil through the application of synthetic nitrogen-based fertilizers mainly fabricated through the Haber-Bosch process. Many processes and microorganisms are sources of N_2_O, being nitrifiers and denitrifiers the two most important groups of soil microorganisms involved (Pilegaard, 2013; Butterbach-Bahl et al., 2014). Denitrification consists in the respiratory reduction of the nitrate present in many terrestrial and aquatic ecosystems. This process is initiated by a periplasmic (Nap) or membrane-bound (Nar) nitrate reductase depending on the species. The nitrite produced from dissimilatory nitrate reduction is then transformed into nitric oxide (NO), a potent cytotoxic free radical and ozone-depleting gas, through the action of the respiratory Cu-containing (NirK) or the *cd*_1_-type nitrite reductase (NirS). Then, NO is reduced to N_2_O by cytochrome *cb*-type (cNor/Cu_A_Nor) or quinol-dependent (qNor) nitric oxide reductases. Finally, a nitrous oxide reductase (Nos) catalyzes the last step of denitrification by producing N_2_ from N_2_O (for recent reviews see de Vries et al., 2007; Zumft and Kroneck, 2007; Richardson, 2011; van Spanning, 2011; Simon and Klotz, 2013; Al-Attar and de Vries, 2015; Torres et al., 2016).

Strategies to mitigate N_2_O emissions from agricultural soils have to be developed in order to decrease current levels of N_2_O production in particular in the context of the continuing population growth (Thomson et al., 2012). One proposed strategy is to promote a sustainable agriculture reducing the dependence on chemical fertilizers and increasing biological nitrogen fixation (BNF) through the application of nitrogen-fixing bacteria to legume crops. However, legumes also contribute to N_2_O emissions by providing N-rich residues into the soils or through the denitrification process that is performed by some rhizobia under free-living or symbiotic conditions (Inaba et al., 2009, 2012; Hirayama et al., 2011; Torres et al., 2016). In fact, many rhizobia species contain denitrification genes. Among them, *Bradyrhizobium diazoefficiens* is considered a model in the study of rhizobial denitrification given its capacity to grow with nitrate as respiratory substrate under anoxic conditions through denitrification, a process that has been extensively investigated in this bacterium (for reviews see Bedmar et al., 2005, 2013; Delgado et al., 2007; Sánchez et al., 2011; Torres et al., 2016). *B. diazoefficiens* denitrification reactions are catalyzed by Nap, NirK, cNor, and Nos enzymes encoded by *napEDABC*, *nirK*, *norCBQD,* and *nosRZDYFLX* genes, respectively. In addition to denitrify, *B. diazoefficiens* is also capable to grow under free-living conditions with nitrate as the sole N source. In this bacterium, the assimilatory nitrate reductase (NasC) constitutes an integrated biochemical system involved in nitrate assimilation and NO detoxification that has been demonstrated to be another source of NO and probably of N_2_O (Cabrera et al., 2016).

*Rhizobium etli* CFN42, the endosymbiont of common bean (*Phaseolus vulgaris*) contains a chromosome and six large plasmids named from pCFN42a to pCFN42f (González et al., 2006). In this bacterium, genes encoding the NirK and cNor denitrification enzymes have been identified on plasmid pCFN42f (Gómez-Hernández et al., 2011). However, genes encoding either a respiratory nitrate reductase (Nar or Nap) or the nitrous oxide reductase enzyme (Nos) are not present in *R. etli* CFN42 genome. Consequently, this rhizobium species is unable to respire nitrate and to perform complete denitrification pathway. Genetic and functional characterization of the reductases encoded by *R. etli nirK* and *norC* suggest a detoxifying role for these enzymes. In fact, phenotypic characterization of *R. etli nirK* and *norC* mutants demonstrated that NirK is required for nitrite reduction to NO and that cNor is required to detoxify NO (Bueno et al., 2005; Gómez-Hernández et al., 2011). Under symbiotic conditions, recent analyses of the levels of nitrosylleghemoglobin complexes (LbNO) of the nodules from common bean plants exposed to nitrate clearly demonstrated the capacity of the nodules to produce NO from nitrate present in the nutrient solution (Gómez-Hernández et al., 2011; Calvo-Begueria et al., 2018). However, the capacity of *R. etli* to produce NO or N_2_O from nitrate under free-living conditions has not been investigated so far. As mentioned before, *R. etli* lacks genes encoding the respiratory nitrate reductases (Nap or Nar). Sequence analysis revealed that an open reading frame in the *R. etli* chromosome (RHE_CHO1780) encodes a putative assimilatory nitrate reductase (NarB). RHE_CHO1780 resides within a cluster of other uncharacterized ORFs (RHE_CHO1781 and RHE_CHO1782) predicted to encode components (NirD and NirB) of an assimilatory nitrite reductase. This genomic context suggests a potential involvement of NarB in nitrate reduction to nitrite that would be further reduced to amonia by NirBD. However, the functional role of *R. etli* NarB has not been studied to date. Through the phenotypic characterization of a *R. etli narB* mutant, in this work we demonstrate the dual role of NarB in nitrate assimilation and in denitrification.

**Table 1:** Bacterial strains and plasmids.

Strain or plasmid	Relevant characteristics	References
**Bacteria**		
*Rhizobium etli*		
CFN42	Nal^r^ (wild-type)	Quinto et al., 1982
CE3	Sm^r^ derivative of CFN42, Nal^r^Sm^r^ (wild-type)	Noel et al., 1984
CFNX702	CE3 derivative, *nirK*::*lox*P, Nal^r^Sm^r^	Gómez-Hernández et al., 2011
CFNX701	CE3 derivative, *norC*::*lox*Sp, Nal^r^Sm^r^ Sp^r^	Gómez-Hernández et al., 2011
DR4000	CE3 derivative, Δ*narB*::ΩSpSm, Nal^r^Sm^r^Sp^r^	This work
*Escherichia coli*		
DH5α	*supE*44Δ*lacU*169 (φ80*lacZ*ΔM15) *hsd*R17*rec*A1*end*A1*gyrA*96 *thi*-1*relA*1	Sambrook et al., 1989
S17.1	*thi, pro*, recA,*hsdR, hsdM*, RP4Tc::Mu, Km::*Tn*7, Tp^r^Sm^r^Sp^r^	Simon et al., 1983
**Plasmids**		
pBluescript KS	Cloning vector, Ap^r^	Invitrogen
pK18*mobsacB*	Suicide cloning vector, Km^r^	Schäfer et al., 1994
pBBR1MCS-2	Broad host range cloning vector, Km^r^	Kovach et al., 1995
pHP45Ω	Vector carrying an ΩSpSm casette	Prentki and Krisch, 1984
pRK415	Broad host range plasmid, Tc^r^	Keen et al., 1988
pNIC-01	pBBMCS53 derivative *norC*::*uid*A, Gm^r^	Gómez-Hernández et al., 2011
pNIC-03	pBBMCS53 derivative *nirK*::*uid*A, Gm^r^	Gómez-Hernández et al., 2011
pDR4000	pK18*mobsac*B carrying *narB* with 2287 bp delection and *narB*::Ω insertion, Sm^r^Sp^r^Km^r^	This work
pDR4002	pBBR1MCS-2 derivative carrying *R. etli narB*, Km^r^	This work
pLG4002	pRK415 derivative carrying *R. etli narB*, Tc^r^	This work

## Materials and Methods

### Bacterial Strains, Plasmids, and Growth Conditions

The bacterial strains and plasmids used in this work are listed in Table 1. *Rhizobium etli* strains were grown at 30°C in TY rich medium (Tryptone Yeast, Beringer, 1974) or in Y minimal medium (MMY) with succinate (10 mM) and ammonium chloride (10 mM) as carbon and nitrogen sources, respectively (Bravo and Mora, 1988). For growth under microoxic or anoxic conditions, flasks containing cell cultures were sealed with rubber septa, and flushed at the starting point of the incubation with 2% (v/v) O_2_ and 98% N_2_ (v/v) or 100% (v/v) N_2_, respectively. For growth with different nitrogen sources, cells were incubated in MMY with 10 mM ClNH_4_, KNO_3_ or NaNO_2_ as sole N source. Antibiotics were added to *R. etli* CE3, and *narB*, *nirK*, and *norC* cultures (see Table 1) at the following concentrations (μg ml^−1^): nalidixic acid (Nal) 20, kanamycin (Km) 30, spectinomycin (Sp) 100, streptomycin (Sm) 100. *Escherichia coli* DH5α used as receptor in cloning experiments and S17.1 used as donor in conjugation experiments were grown at 37°C in LB medium (Sambrook and Russell, 2001) and the antibiotics were added at the following concentrations (μg ml^−1^): spectinomycin 25, streptomycin 25, kanamycin 20, and ampicillin 200.

For determination of growth rates and enzymatic activities, cells were firstly grown aerobically in TY medium for 24 h, harvested by centrifugation at 8000 *g* for 10 min and washed twice with MMY containing ClNH_4_, KNO_3_ or NaNO_2_ as sole N source, depending on the treatment. Then, cells were incubated in the minimal medium for another 24 h under the desired oxygen conditions. Initial optical density at 600 nm of the cultures was around 0.05 for growth rates measurements or around 0.25 for enzymatic activity analyses.

For characterization of *narB* mutant growth, an additional step under starvation conditions was included before growing cells in the minimal medium. The starvation step consisted of a 24 h incubation of the cells in the minimal medium containing salts (CaCl_2_ and FeCl_3_), and lacking any nitrogen or carbon sources.

### Construction and Complementation of *R. etli narB* Mutant

The oligonucleotide primers (Sigma) used in this work are listed in Supplementary Table S1. Genomic and plasmid DNA isolation were carried out using the REALPURE Genomic DNA purification Kit (Real) and Qiagen Plasmid Kit (Qiagen), respectively. PCR was performed using the High Fidelity DNA polymerase Phusion^®^enzyme (Thermo Fisher Scientific) and DNA digestions were carried out using Fast digest enzymes (Thermo Fisher Scientific).

To generate the *narB* mutant, the two regions flanking the *narB* gene (fragments F1 and F2), were amplified by PCR using narB_up_For/narB_up_Rev (in positions 1864715 to 1864732 and 1865293 to 1865310, respectively) and narB_down_For/narB_down_Rev (in positions 1867579 to 1867598 and 1868201 to 1868220, respectively) primer pairs. Then, fragments F1 and F2 were cloned into the pBlueScriptKS (pBSKS) vector (Invitrogen) as XbaI/BamHI and BamHI/EcoRI fragments, respectively, generating plasmid pBKS_F1F2. To construct a suicide plasmid useful for double recombination, the XbaI/EcoRI fragment from pBKS_F1F2 was cloned into the pK18*mobsacB* suicide vector (Schäfer et al., 1994) yielding plasmid pK18F1F2. This plasmid was further modified by inserting the ΩSp/Sm cassette (Prentki and Krisch, 1984) into the BamHI site of pK18F1F2 plasmid (between F1 and F2 fragments) obtaining plasmid pDR4000 that was analyzed by sequencing. Replacement of the *R. etli narB* wild type allele with the truncated mutant allele in plasmid pDR4000 was carried out by double recombination. With this purpose, plasmid pDR4000 was transferred via conjugation into *R. etli* CE3 using *E. coli* S17-1 as donor. Double recombination events were favored by growth on agar plates containing sucrose using the *sacB* marker present in plasmid pK18*mobsacB*. Double recombinants were selected as resistant to Sm and Sp and susceptible to Km. To verify that the gene replacement had occurred, the derivatives were analyzed by PCR using primers narB_EXT-For, narB_EXT-Rev, narB_IN-For, and narB_IN-Rev (Supplementary Table S1) and the *narB* mutant strain was named DR4000.

A plasmid carrying the *R. etli narB* gene constitutively expressed from the *lacZ* promoter was obtained by cloning the *narB* coding region in the broad-host range cloning vector pBBR1MCS-2 (Kovach et al., 1995). To that end, the *narB* gene was amplified by PCR using narB_compl_For/narB_compl_Rev primers (Supplementary Table S1) and cloned as an XbaI/HindIII fragment into pBBR1MCS-2. The plasmid obtained (pDR4002) was sequenced and transferred to *R. etli* CE3 (WT) and DR4000 strains by conjugation using *E. coli* S17-1 as donor. The strain derivatives containing pDR4002 (WT/pDR4002 and DR4000/pDR4002, respectively) were checked by plasmid isolation and PCR. Concurrently, a WT strain containing pBBR1MCS-2 empty vector was obtained (WT/pBBR1MCS-2), as a control.

An additional plasmid carrying the *narB* gene constitutively expressed from the *lacZ* promoter was constructed by cloning the XbaI/HindIII fragment containing *narB* from pDR4002 into the pRK415 vector (Keen et al., 1988). The plasmid obtained (pLG4002) was introduced by conjugation into *R. etli* DR4000 strain harboring plasmids pNIC-03 and pNIC-01 that contain a *nirK*::*uidA* or *norC::uidA* transcriptional fusions, respectively. In addition, the pRK415 empty vector was introduced into WT- pNIC-03, WT- pNIC-01, DR4002- pNIC-03, and DR4002- pNIC-01 derivatives.

### Extracelullar NO_2_^−^ Determination

To measure the concentration of NO_2_^−^ in the medium during growth with NO_3_^−^ under aerobic or microoxic conditions, aliquots were taken from cultures at different time points. Culture samples were centrifuged at 8000 *g* for 10 min and nitrite concentration was estimated in the supernatant after diazotization by adding the sulphanilamide-naphthylethylenediamine dihydrochloride reagent (Nicholas and Nason, 1957).

### Cell Extract Preparation and Determination of Nitrate and Nitrite Reductase Activities

To analyze nitrate reductase (NR) activity, cells at an initial OD_600_ of about 0.25 were incubated aerobically with KNO_3_ as the sole nitrogen source for 24 h. After centrifugation at 8000 g for 10 min, cells were harvested and disrupted by using a French pressure cell (SLM Aminco, Jessup, MD, United States). Then, fractionated cells were centrifuged at 10000 *g* for 10 min and the supernatant containing the soluble cell extract was used for NR activity. To analyze nitrite reductase (Nir) activity, cells were grown microoxically with KNO_3_ as the sole nitrogen source for 24 h. Then, cells were harvested by centrifugation, washed twice with 50 mM Tris–HCl pH 7.5 and resuspended in 1 ml of the same buffer.

Methyl-viologen dependent nitrate reductase (MV-NR) and nitrite reductase (MV-Nir) activities were determined by using 105 μl of the soluble cell extract (∼0.5 mg protein) or cell suspension (∼0.1 mg protein), respectively. The reaction mixture also contained 0.2 mM Methyl Viologen and 10 mM KNO_3_ for NR activity or 0.01 mM NaNO_2_ for Nir activity. The reaction was started by the addition of 15 μl of freshly prepared 144 mM sodium dithionite solution in 300 mM NaHCO_3_. After incubation for 20 min at 30°C, the reaction was stopped by vigorous shaking until the samples had lost their blue color. Nitrite produced by NR or consumed by Nir enzymes was estimated after diazotization as described previously for extracellular NO_2_^−^ determination.

### NO Production and Consumption Activities

In order to investigate the capacity of the different mutants to produce or consume NO, cell cultures at an initial OD600 of about 0.25 were incubated microoxically with KNO_3_ as the sole nitrogen source for 24 h, harvested by centrifugation, washed twice with 25 mM Na_2_HPO_4_/NaH_2_PO4 buffer (pH 7.4), and resuspended in 1.5 ml of the same buffer. NO production and consumption activities were determined by using an ISONOP NO electrode APOLLO 4000^®^(World Precision Instruments). The reaction chamber (2 ml) was temperature-controlled, magnetically stirred and contained: 1410 μl of 25 mM Na_2_HPO_4_/NaH_2_PO_4_ buffer (pH 7.4) and 250 μl of cell suspension (0.4–0.7 mg protein) for NO production or 760 μl of 25 mM Na_2_HPO_4_/NaH_2_PO_4_ buffer (pH 7.4) and 900 μl of cell suspension (1.5–2.5 mg protein) for NO consumption. To generate an anoxic atmosphere, 100 μl of an enzymatic mix containing *Aspergillus niger* glucose oxidase (40 units⋅ml^−1^), bovine liver catalase (250 units⋅ml^−1^) (Sigma-Aldrich), 90 μl of 1 M sodium succinate and 100 μl of 320 mM glucose were added to the chamber. Once a steady base line was obtained, 50 μl of 50 mM NaNO_2_ (NO production) or 50 μl of 2 mM NO (NO consumption) was added to the chamber to start the reaction.

### N_2_O Production

To measure N_2_O accumulation, *R. etli* CE3 and the mutant strains were cultured as indicated above for NO experiments. After 24 h growth, 1 ml was taken from the headspace of cultures, using a Hamilton^®^Gastight syringe, and manually injected into an HP 4890D gas chromatography instrument equipped with an electron capture detector (ECD) as described by Torres et al. (2014).

### Haem-Staining Analysis

To study the expression of the NorC component of cNor, we performed haem *c*-staining analyses of proteins from membranes of *R. etli* CE3 and the mutant strains cultured as indicated above for NO and N_2_O experiments. After 24 h growth, cells were harvested by centrifugation, washed twice with 50 mM Na_2_HPO_4_/NaH_2_PO_4_ (pH 6.8) buffer containing 1 mM MgCl_2_, 0.9% NaCl and 0.1 mM CaCl_2_, and resuspended in 2.5 ml of the same buffer containing 0.1 mM 4-(2-aminoethyl) benzene-sulfonyl fluoride hydrochloride (ABSF), RNase (20 *μ*g ml^−1^), and DNase I (20 *μ*g ml^−1^). Cells were disrupted using a French pressure cell (SLM Aminco, Jessup, MD, United States) and the membrane fraction was prepared as described previously (Torres et al., 2013). Then, membrane proteins were separated by SDS-PAGE, transferred to a nitrocellulose membrane and stained for haem-dependent peroxidase activity as described previously (Vargas et al., 1993) using the chemiluminescence detection kit “SuperSignal” (Thermo Fisher Scientific, Pierce, IL, United States). Protein concentration was estimated using the Bio-Rad assay (Bio-Rad Laboratories).

### Measurement of β-Glucuronidase Activity

To analyze the expression of *nirK* and *nor* genes, *R. etli* CE3 and the mutant cells containing a *nirK::uidA* or a *norC::uidA* transcriptional fusions were incubated microoxically (2% initial O_2_ concentration) for 14 h in MMY medium containing ClNH_4_ or KNO_3_ as sole N source, with exception of the *narB* mutant that was grown for 21 h. Quantitative GUS activity was determined on 1.0-ml culture samples using 4-nitrophenyl β-D-glucuronide as substrate as described previously (Girard et al., 2000). Data were normalized to total cell protein concentration by the Lowry method over a second set of 1.0-ml samples.

## Results

### *R. etli narB* Encodes the Assimilatory Nitrate Reductase

**FIGURE 1:**
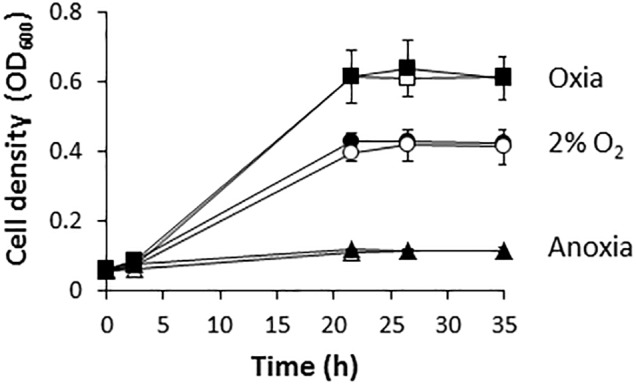
Nitrate-dependent growth of *R. etli*. Cells were cultured under oxic (squares), microoxic (circles) or anoxic (triangles) conditions, with either NH_4_^+^ (white) or NO_3_^−^ (black), as sole N source. Results are the mean values and error bars from at least two different cultures assayed in triplicate.

Figure 1 shows that *R. etli* (WT) is able to grow with NO_3_^−^ as the sole nitrogen source under oxic or microoxic (2% initial O_2_ concentration) conditions reaching values of optical densities (OD) at 600 nm of around 0.6 or 0.4, respectively. However, this bacterium was unable to use NO_3_^−^ for respiration being incapable to grow under anoxic conditions with nitrate as the sole N source (Figure 1). These results suggest that *R. etli* uses nitrate through the assimilatory pathway under oxic or microoxic conditions where oxygen was used for respiration. However, it is unable to respire nitrate when oxygen is absent. Similar growth rates were reached when the wild-type (WT) cells were grown in minimal medium amended with 10 mM of ClNH_4_ as the sole N source (Figure 1).

**FIGURE 2:**
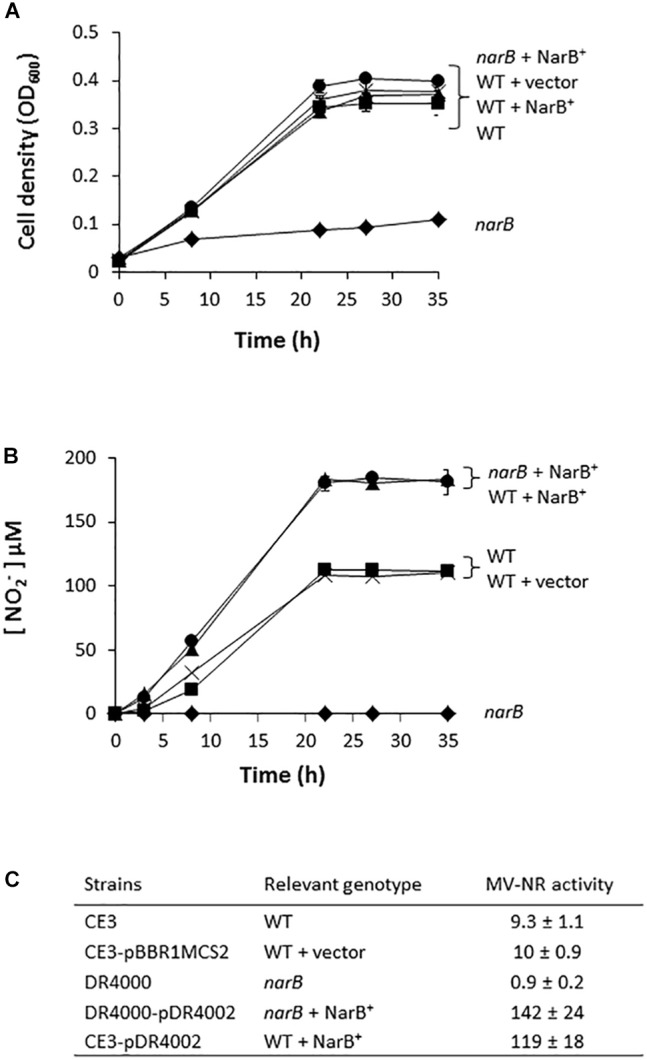
Nitrate-dependent aerobic growth and NR activity of a *R. etli narB* mutant. **(A)** Growth curves. **(B)** Extracellular nitrite concentration in the growth medium. **(C)** MV-NR activity expressed as nmol NO_2_^−^ produced mg protein^−1^ h^−1^. *R. etli* WT (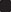), WT + vector (X), *narB* (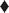), *narB* + NarB^+^ (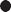), and WT + NarB^+^ (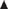) strains were incubated aerobically in minimal medium with NO_3_^−^ as sole N-source. Data are expressed as the mean value ± SD from two different cultures assayed in triplicate.

In order to determine the implication of RHE_CHO1780 encoding for a putative assimilatory nitrate reductase (NarB) in nitrate assimilation, a *R. etli* mutant strain lacking the *narB* gene was constructed. As shown in Figure 2A, aerobic growth of *R. etli narB* mutant was highly decreased compared to that reached by the WT strain (0.1 and 0.4 OD_600_, respectively, after 35 h culture). However, when the *narB* mutant was complemented with plasmid pDR4002 that constitutively expresses *narB* (*narB* + NarB^+^), similar growth rates as those from WT cells were observed (Figure 2A). No significant differences of growth rates were found when the WT strain was complemented with plasmid pDR4002 (WT + NarB^+^) or with pBBR1MCS-2 (WT + vector) (Figure 2A). Moreover, NO_2_^−^ was not detected in the culture medium of *narB* mutant grown oxicallly (Figure 2B). However, WT cells accumulated around 100 μM NO_2_^−^ in the medium after 35 h growth (Figure 2B). Interestingly, both *narB* and WT strains containing pDR4002 (*narB* + NarB^+^ or WT + NarB^+^) accumulated about 180 μM NO_2_^−^ in the medium. These results suggest that *R. etli* NarB is the enzyme responsible for nitrate reduction to nitrite, the first step of nitrate assimilation. To corroborate this observation, we also measured MV-NR activity of *R. etli* cells grown under oxic conditions with NO_3_^−^ as sole N source. As shown in Figure 2C, MV-NR activity was around 10-fold lower in the *narB* mutant compared to that observed in the WT strain. The constitutive expression of *narB* in the *narB* mutant (*narB* + NarB^+^), restored NR activity to levels significantly higher (about 15-fold) to those observed in WT cells. A similar increase of NR activity (about 13-fold) was observed in WT cells containing pDR4002 compared to NR levels of WT strain with or without the empty vector pBBR1MCS-2 (Figure 2C). The higher effect of the presence of pDR4002 on nitrite accumulation (Figure 2B) and MV-NR activity (Figure 2C) is due to the over-expression of *narB* gene by the constitutive *lacZ* promoter present in pDR4002. Taken together, these results confirm the participation of NarB in nitrate reduction to nitrite when cells are cultured under aerobic conditions with nitrate as the only N source.

### *R. etli narB*, *nirK*, and *norC* Are Required for Denitrification

**FIGURE 3:**
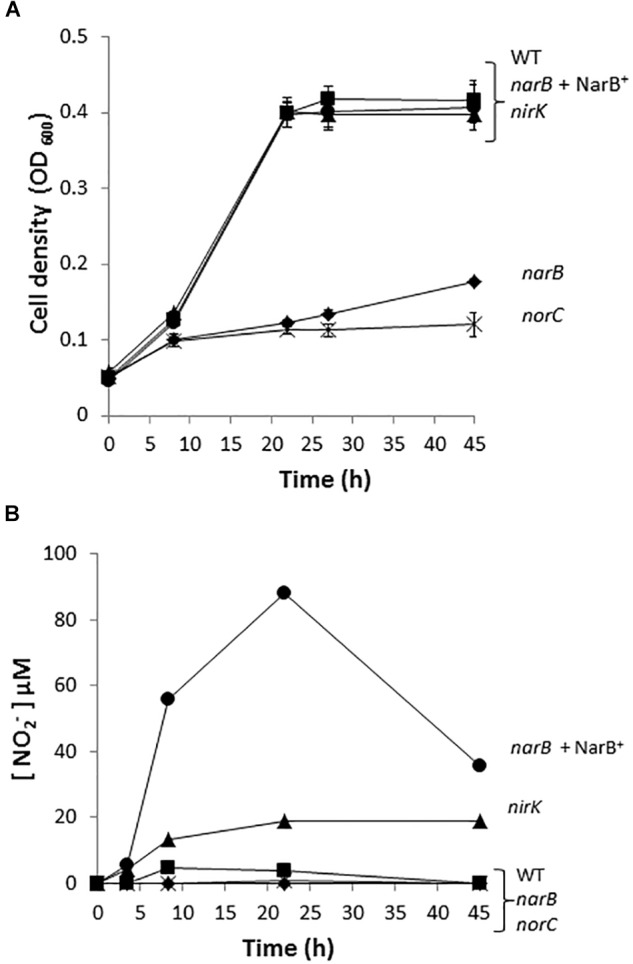
Nitrate dependent micro-oxic growth of *R. etli narB*, *nirK,* and *norC* mutants. **(A)** Growth curves. **(B)** Extracellular nitrite concentration in the growth medium. *R. etli* WT (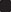), *narB* (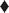), *narB*+ NarB^+^ (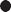), *norC* (X), and *nirK* (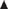) strains were cultured microoxically in minimal medium with NO_3_^−^ as sole N-source. Data are expressed as the mean value and error bars from two different cultures assayed in triplicate.

To investigate the implication of *R. etli narB*, *nirK* and *norC* genes in the denitrification process, we performed growth rate experiments of the *R. etli narB, nirK* and *norC* mutant cells cultured under microoxic conditions and in the presence of nitrate as sole nitrogen source. While *narB* was obtained in this work, the *nirK* or *norC* mutants were previously constructed by Gómez-Hernández et al. (2011). As observed when cells were incubated under oxic conditions (Figure 1A), the *narB* mutant showed a clear defect in its ability to grow when compare to the WT cells (Figure 3A). Complementation of *narB* mutant with plasmid pDR4002 expressing constitutively *narB* (*narB*+ NarB^+^) restored the WT ability to grow under microoxic conditions (Figure 3A). The *norC* mutant was completely unable to grow microoxically with nitrate as unique nitrogen source. However, no differences in nitrate-dependent growth rates were detected between the *nirK* mutant and WT strain (Figure 3A). The capacity of the *nirK* mutant to grow with nitrate might be due to its ability to assimilate nitrate and nitrite through the activity of the NarB and NirBD assimilatory nitrate and nitrite reductase enzymes. As shown in Figure 3B, WT cells incubated microoxically with NO_3_^−^ accumulated low levels of NO_2_^−^ in the medium after 8 h incubation (5 μM NO_2_^−^) that was consumed after 45 h growth. Growth of the *R. etli nirK* mutant under the same growth conditions resulted in higher levels of NO_2_^−^ concentration in the medium compared to those observed in WT cultures (20 μM *versus* 0 μM NO_2_^−^ after 45 h incubation) (Figure 3B). Furthermore, levels of nitrite detected in the culture medium of *narB* or *norC* mutants cultivated under the same conditions were undetectable (Figure 3B). Interestingly, *narB* mutant containing plasmid pDR4002 (*narB* + NarB^+^) accumulated about 11-times more NO_2_^−^ in the medium compared to WT cells (56 μM *versus* 5 μM NO_2_^−^ after 8 h incubation) (Figure 3B).

**FIGURE 4:**
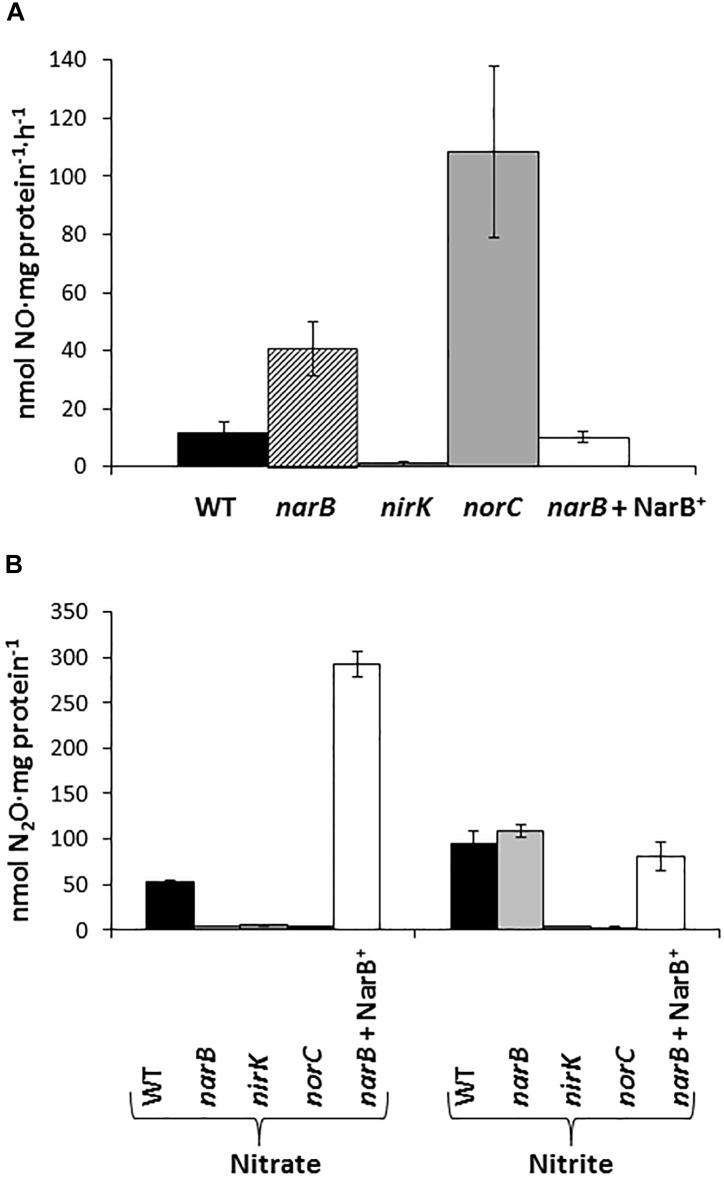
Nitric oxide production capacity **(A)** and N_2_O accumulation **(B)** by *R. etli narB*, *narB* + NarB^+^, *nirK*, and *norC* mutants. In **(A)** cells incubated microoxically with NO_3_^−^ were transferred to a reaction chamber with NO_2_^−^. In **(B)** cells were cultured microoxically with NO_3_^−^ or NO_2_^−^ as the sole N-source. Data are expressed as the mean value and error bars from two different cultures assayed in triplicate.

In order to investigate the denitrification capacity of the *narB*, *nirK* and *norC* mutants, we determined MV-Nir, NO consumption activity (Nor), NO production capacity and N_2_O accumulation (Figure 4 and Table 2). As shown in Table 2, MV-Nir activity was about 5-times lower in the *nirK* mutant compared to that observed in WT, *narB* or *narB* containing pDR4002 (*narB* + NarB^+^) strains. The residual Nir activity observed in the *nirK* mutant could be due to the activity of an assimilatory Nir, enzyme that is also encoded in the *R. etli* CFN42 genome. Concerning Nor activity, it was significantly lower (around ninefold) in cells of the *norC* mutant compared to the values reached by WT cells (Table 2). These observations indicate that NirK and NorC are the main enzymes responsible of MV-Nir and Nor activities, respectively. Furthermore, a significant reduction (around twofold) of Nor activity was observed in the *narB* mutant. On the contrary, the constitutive expression of *narB* in the *narB* mutant background (*narB* + NarB^+^) resulted in an increase of about 3.4-fold of Nor activity compared to that observed in the *narB* mutant (Table 2). Additionally, we measured NO production capacity of *nirK*, *norC*, and *narB* mutants cultured microoxically with nitrate that were transferred to a reaction chamber with NO_2_^−^ as substrate (Figure 4A). The *norC* mutant accumulated about 10-times more NO than the WT being the toxicity of NO the reason that might explain the inability of this mutant to grow under microoxic conditions (Figure 3A, 4A). On the contrary, the *nirK* mutant did not produce NO. NO accumulation capacity by the *narB* mutant was 4-times higher than that observed in the WT strain (Figure 4A) probably due to the twofold reduction of NO consumption activity observed in the *narB* mutant (Table 2) compared to WT cells. Accordingly, NO produced by the *narB* mutant containing pDR4002 (*narB* + NarB^+^) decreased to WT levels (Figure 4A). Interestingly, N_2_O production was observed in the headspace of WT cultures grown under microoxic conditions with nitrate as the only N source (Figure 4B). By contrary, *narB*, *nirK* and *norC* mutants appeared to be unable to produce N_2_O (Figure 4B). However, the *narB* mutant complemented with pDR4002 (*narB* + NarB^+^) showed a significant accumulation (about sixfold) of N_2_O compared to those levels produced by the WT strain in the presence of nitrate in the growth medium. These results clearly demonstrate the involvement of nitrate reduction by NarB on *R. etli* N_2_O emission in cells grown with nitrate. However, when cells were grown in the presence of nitrite as the sole nitrogen source, the *narB* mutant as well as the *narB* + NarB^+^ strain reached similar values of N_2_O accumulation to the WT (Figure 4B). As observed in nitrate-cultured cells, *nirK* and *norC* mutants were defective in their capacity to produce N_2_O in cells with NO_2_^−^ as N source (Figure 4B). These results indicate that NirK and NorC but not NarB are required for N_2_O production by *R. etli* cells cultured with nitrite as N source.

**Table 2:** MV-Nir and Nor activities of *R. etli narB*, *nirK*, *norC*, or *narB* complemented with pDR4002 (NarB^+^).

		Activities	
Strains	Genotype	MV-Nir	Nor
CE3	WT	195 ± 2.3	269 ± 58
DR4000	*narB*	182 ± 1.6	155 ± 43
CFNX702	*nirK*	40 ± 2.3	nd
CFNX701	*norC*	nd	31 ± 5.4
DR4000-pDR4002	*narB* + NarB^+^	190 ± 4.5	532 ± 72

*Cells were cultured microoxically with NO_3_^−^ as the sole N-source. MV-NiR and Nor activities are expressed as nmol of NO_2_^−^ or NO consumed⋅h^−1^⋅mg protein^−1^, respectively. Data are expressed as the mean value ± SD from at least two different cultures assayed in triplicate; nd, not determined.*

**Table 3:** β-glucuronidase specific activity of *nirK*::*uidA* and *norC*::*uidA* transcriptional fusions in *R. etli* WT, *narB*, or *narB* complemented with pLG4002 (NarB^+^).

	β-glucuronidase specific activity^1^		
Strains	Genotype	NH_4_^+^	NO_3_^−^
CE3-pRK415-pNIC-03	WT (*nirK*::*uidA*)	1115 ± 72	2087 ± 106
CE3-pRK415-pNIC-01	WT (*norC*::*uidA*)	352 ± 51	1451 ± 185
DR4000-pRK415-pNIC-03	*narB (nirK*::*uidA*)	912 ± 17	1617 ± 131
DR4000-pRK415-pNIC-01	*narB* (*norC*::*uidA*)	245 ± 6	223 ± 8
DR4000-pLG4002-pNIC-03	*narB*+NarB^+^ (*nirK*::*uidA*)	1236 ± 75	2356 ± 122
DR4000-pLG4002-pNIC-01	*narB+*NarB^+^ (*norC*::*uidA*)	453 ± 58	1493 ± 108
CFNX702- pNIC-01	*nirK (norC::uidA)*	91 ± 33	119 ± 23

*Cells were cultured microoxically with NH_4_^+^ or NO_3_^−^ as the sole N source. ^1^Values are expressed as nmol min^−1^mg protein^−1^. Data are the mean of three replicates from two independent experiments ± SD.*

### Involvement of Nitrate Reduction by NarB in *R. etli nirK* and *nor* Expression

Results from Table 2 suggest the involvement of NarB in Nor activity, but not in Nir activity. Our next goal was to evaluate the participation of NarB in the expression of *nirK* and *nor* genes. To achieve this goal, a *nirK::uidA* and a *norC::uidA* transcriptional fusions present in plasmids pNIC-03 or pNIC-01, respectively (Gómez-Hernández et al., 2011) were used in this work. *R. etli* WT cells grown with NO_3_^−^ showed a slight increase of about twofold of *nirK::uidA* expression compared to that from NH_4_^+^-grown cells (Table 3). However, about fourfold increase of *norC::uidA* was observed in NO_3_^−^-grown cells compared to those grown with NH_4_^+^ as N source (Table 3). Interestingly, a differential dependence on NarB for expression of *nirK* and *norC* genes was observed when NO_3_^−^ was present in the culture medium. While *nirK* showed only a marginal dependence of NarB for expression in this condition (2087 ± 106 in the WT vs. 1617 ± 131 in the *narB* mutant), the fourfold induction of *norC* expression by nitrate in the WT was not observed in the *narB* mutant (Table 3). As shown in Table 3, the expression of *norC* in the *narB* mutant strain was restored when it contained plasmid pLG4002 with a constitutive expression of *narB* (223 ± 8 to 1493 ± 108 activity values). By contrast, the presence of pLG4002 in the *narB* mutant slightly increased *nirK::uidA* expression in NO_3_^−^-grown cells (1617 ± 131 to 2356 ± 122 activity units). These results clearly show that the induction of the microoxic expression of *norC* by nitrate is dependent on NarB. We also observed that the induction of the *norC::uidA* expression in response to nitrate did not occur in a *nirK* mutant background (Table 3). These results suggest that NO produced from NO_3_^−^ reduction to NO_2_^−^ by NarB and from NO_2_^−^ reduction by NirK is the nitrogen oxide (NOx) required for *nor* expression.

**FIGURE 5:**
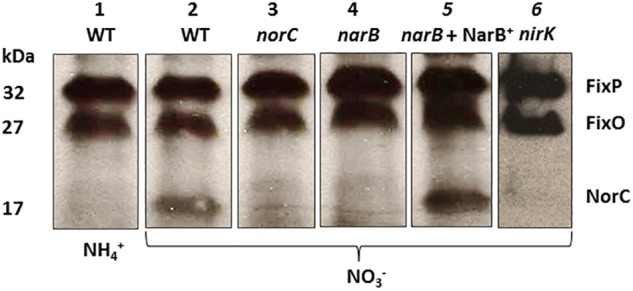
Expression of NorC in *R. etli narB* mutant. Haem-stained proteins of membranes prepared from *R. etli* WT (lanes 1 and 2), *norC* (lane 3), *narB* (lane 4), *narB* + NarB^+^ (lane 5), and *nirK* (lane 6) mutants cultured microoxically with NH_4_^+^ (lane 1) or NO_3_^-^ (lanes 2, 3, 4, 5, and 6) as the sole N-source. Each lane contains 25 μg of membrane proteins. Haem-stained *c*-type cytochromes indentified previously (FixP and FixO) and in this work (NorC) are indicated. Apparent protein molecular masses (kDa) are also shown.

To confirm the participation of NarB in the induction of *nor* genes, we examined the expression of NorC by performing haem *c* staining analyses in proteins from membranes of a *narB* mutant cultured microoxically with nitrate as N source. To identify NorC protein, we also included in these experiments the *R. etli norC* mutant. As shown in Figure 5, a 32- and 27-kDa *c*-type cytochromes, identified previously as the FixP and FixO subunits of the terminal oxygen high-affinity *cbb*_3_-type cytochrome oxidase (Soberon et al., 1999) were observed in all strains. A third band of about 17 kDa which was observed in WT cells grown with NO_3_^−^ could not be detected in membranes from the *norC* mutant cultured under the same conditions (Figure 5, lanes 2 and 3). These results allowed us to identify by the first time in *R. etli* the NorC component of cNor. By contrary to WT nitrate-dependent cells, growth with NH_4_^+^ as N source did not allowed expression of NorC suggesting the requirement of nitrate in the medium to induce NorC in *R. etli* (Figure 5, lanes 1 and 2). Interestingly, NorC could not be detected in membranes from the *narB* mutant grown with nitrate indicating that nitrate reduction by NarB, is required to induce NorC expression in *R. etli* (Figure 5, lanes 2 and 4). In fact, constitutive expression of NarB in the *narB* mutant (*narB* + NarB^+^) restored the expression of NorC in *R. etli narB* mutant cultured microoxically with nitrate (Figure 5, lanes 2, 4, and 5). These results clearly demonstrate the role of nitrate reduction by NarB in NorC expression in *R. etli*. In this bacterium, the reduction products of nitrate under microoxic conditions are NO_2_^−^ or NO. In order to identify the NOx (NO_2_^−^ or NO) required for expression of NorC, we included in the haem-staining experiments the *nirK* mutant which does not produce NO from nitrate (Figure 4A). As shown in Figure 5 (lane 6), NorC was not detected in membranes from the *nirK* mutant in response to nitrate, suggesting that NO could be the signal molecule implicated in NorC expression.

## Discussion

### *R. etli* NarB Is Required for Nitrate Assimilation and Denitrification

*Rhizobium etli* is a N_2_-fixing soil bacterium able to establish endosymbiotic associations with common bean plants. Up to now, the capacity of this bacterium to perform other processes of the N-cycle was unknown. In this work, we have demonstrated for the first time the ability of *R. etli* to assimilate nitrate as well as to produce NO and N_2_O from NO_3_^−^ through denitrification. However, *R. etli* is unable to grow under anoxic conditions using NO_3_^−^ as the final electron acceptor since it lacks the respiratory Nap or Nar nitrate reductases. The denitrification enzymes NirK and cNor are not proton pump or electrogenic. In this case, is the electron transfer from UQ pool to *bc*_1_ complex that can be used to drive the translocation of protons across the mitochondrial membrane to generate a *trans*-membrane proton electrochemical gradient or proton motive force (Δp) that can drive the synthesis of ATP. However, in incomplete denitrifiers like *R. etli* CFN42 that lack Nar or Nap, ATP synthesis from electron transfer to NirK and cNor is limited and it does not allow cells to grow from nitrate respiration anoxically. An inspection of the *R. etli* CFN42 genome^1^, allowed us to identify a gene (RHE_CHO1780) annotated as *narB*, which encodes a putative NarB. Two classes of assimilatory nitrate reductases (Nas) have been described from microorganisms: the NADH-dependent Nas and the ferrodoxin- or flavodoxin-dependent Nas (Fd-Nas, Moreno-Vivian et al., 1999). NADH-Nas proteins are heterodimers consisting of a 45 kDa FAD-containing diaphoarase and the 95 kDa catalytic subunit with a molybdenum *bis*-molybdopterin dinucleotide (Mo-*bis*-MGD) cofactor and a N-terminal [4Fe-4S] center (Richardson et al., 2001). Fd-Nas usually present in cyanobacteria are monomers with a molecular weight between 75 and 85 kDa (Moreno-Vivián and Flores, 2007). The *in silico* analysis of the *R. etli* NarB sequence (web.expasy.org) found that it has 885 aa, a predicted molecular weight of approximately 94.5 kDa, as well as the typical Mo-*bis*-MGD binding domain and the consensus motifs for co-ordination of an N-terminal [4Fe-4S] cluster present in NADH-Nas.

In nitrate assimilation, NO_3_^−^ is incorporated into the cells by its sequential reduction to NO_2_^−^ and NH_4_^+^ by the assimilatory nitrate and nitrite reductases, respectively. The presence of NarB in *R. etli* led us to hypothesize that this bacterium could use this enzyme to assimilate nitrate together with the two additional ORFs located in the same gene cluster that are predicted to encode the NirB and NirD components of an assimilatory nitrite reductase. The NH_4_^+^ produced is further incorporated into carbon skeletons (reviewed by Luque-Almagro et al., 2011). In fact, a *R. etli narB* mutant was unable to grow aerobically with nitrate as the only N source and was impaired in nitrate reductase activity.

*Rhizobium etli* CFN42 only possesses in its genome the *nirK* and *nor* denitrification genes, encoding NirK and cNor, respectively. Previous results have demonstrated that NirK is required for nitrite reduction to NO and that cNor is needed to detoxify NO (Bueno et al., 2005; Gómez-Hernández et al., 2011). However, up to now, no evidence has been reported about the putative link between NarB and nitrite and NO detoxification in nitrate-dependent microoxically grown cells. In this work, we have demonstrated that NarB is required for N_2_O formation in cells grown microoxically with nitrate as N source given the inability of the *R. etli narB* mutant to produce N_2_O from nitrate. These observations led us to suggest that NO_2_^−^ produced by NO_3_^−^ reduction in the cytoplasm through NarB activity might be exported outside the cell where is detoxified by NirK and cNor to produce NO and N_2_O, respectively, in the periplasmic space. In the same genomic region where NarB is located, there is an ORF (RHE_CHO1783) that is predicted to encode a major facilitator family NO_3_^−^/NO_2_^−^ transporter (NarK) similar to that found in *B. diazoefficiens* that has been reported to be involved in NO_2_^−^ extrussion (Cabrera et al., 2016). The implication of this NarK-like protein in transporting NO_2_^−^ from the cytoplasm to the periplasm is under investigation. The involvement of NarB in nitrate assimilation and denitrification was also demonstrated by complementing the *narB* mutant with the constitutively expressed *narB* gene allowing the restoration of the ability to assimilate nitrate and to produce N_2_O in the *narB* mutant. Interestingly, the constitutive expression of *narB* in the *narB* mutant resulted in a remarkable increase of NR activity, NO_2_^−^ accumulation in the medium and Nor activity that resulted in higher levels of N_2_O respect to WT cells under microoxic conditions. However, this increase in nitrate reduction and N_2_O formation did not result in higher growth rates probably due to the fact that NarB, NirK, and cNor enzymes do not allow the cells to obtain energy through the assimilative NO_3_^−^ reduction by NarB coupled to denitrification by NirK and cNor. In fact, constitutive expression of NarB in *R. etli* WT strain did not allow the cells to grow under anoxic conditions with nitrate (data not shown). On the contrary, it has been recently reported that constitutive expression of Nap allowed *Ensifer meliloti* to increase the production of NO_2_^−^, NO, and N_2_O as well as its capacity to grow anoxically using nitrate as respiratory substrate (Torres et al., 2018). In spite of *R. etli narB*, *nirK*, and *norC* genes do not allow cell growth through denitrification, when cells are cultured microoxically through nitrate assimilation, *nirK*, and *norC* have a detoxifying role preventing the accumulation of the cytotoxic molecules nitrite and NO and contributing to the production of the GHG N_2_O having an environmental impact to climate change. In this context, the role of *R. etli* NirK and cNor on nitrite and NO detoxification has been previously reported (Gómez-Hernández et al., 2011).

### Nitrate-Dependent Induction of *R. etli nor* Expression Requires NarB

*Bradyrhizobium diazoefficiens*, the symbiont of soybeans, is considered a model for the study of denitrification in rhizobia given its capacity to grow anoxically from nitrate respiration. In this bacterium, where denitrification has been extensively studied, expression of *nap*, *nirK*, *nor*, and *nos* genes requires both oxygen limitation and the presence of a NOx (for a recent review see Torres et al., 2016). In *R. etli*, it has been reported that low-oxygen concentration (1%) induces expression of *nirK* and *norC* denitrification genes (Gómez-Hernández et al., 2011). In this work, we demonstrate that in addition to low oxygen conditions, nitrate or a product derived from its reduction generated by NarB activity is also required for induction of *nor* genes but not for *nirK*. In fact, Gómez-Hernández et al. (2011) found that *R. etli norC* and *nirK* genes display a different level of dependence for the transcriptional regulator NnrR. A null mutation in *nnrR* causes a drastic drop in the expression of *norC*, while *nirK* still exhibits significant expression. In *B. diazoefficiens*, NnrR is the direct transcriptional regulator of *nor* genes in response to NO but not of *nirK* that is controlled directly by FixK_2_ in response to low oxygen (Bueno et al., 2017). These findings are in agreement with the different dependency on nitrate and NarB of the *R. etli nor* and *nirK* genes we show in this work.

In order to identify the NOx (NO_2_^−^ or NO) derived from nitrate reduction required for induction of *R. etli nor* genes, we analyzed the expression of the *norC::uidA* transcriptional fusion as well as the haem *c* component of cNor (NorC) in a *R. etli nirK* mutant that is defective in nitrite reduction to NO as it has been demonstrated in this work. The absence of NorC in membranes as well as the very basal levels of β-glucuronidase activity in the *nirK* mutant cultured with nitrate as sole N source indicates that the signal molecule required for induction of *nor* genes is NO. Similarly, it has been recently demonstrated in *B. diazoefficiens* that *norCBQD* expression requires, in addition to microoxia, the presence of NO (Bueno et al., 2017).

In this work, we propose for the first time a new pathway in bacteria to produce N_2_O by coupling nitrate assimilation and denitrification under microoxic conditions. In this context, it has been recently identified in *B. diazoefficiens,* an integrated system for nitrate assimilation and nitric oxide detoxification which is connected to denitrification through the induction of *nor* genes when a single domain hemoglobin (Bjgb) encoded in this pathway is not functional (Cabrera et al., 2016).

## Author Contributions

AH-G and MD conceived and designed the study. AH-G, MT, AS, and LG performed the experiments. AH-G, MT, AS, LG, and MD analyzed the results and wrote the manuscript. EB critically revised the manuscript. All authors read and approved the final manuscript.

## Conflict of Interest Statement

The authors declare that the research was conducted in the absence of any commercial or financial relationships that could be construed as a potential conflict of interest.

## Footnotes

1 http://genome.microbedb.jp/rhizobase
