# Supplementary material for: Rhizobium etli Produces Nitrous Oxide by Coupling the Assimilatory and Denitrification Pathways
Source: Front Microbiol. 2019 May 7;10:980. doi: 10.3389/fmicb.2019.00980 (PMC6514139; doi:10.3389/fmicb.2019.00980)
Supplement: Supplementary file 1 [file Table_1.DOCX]

Table S1. Oligonucleotide primers used in this work. Restriction sites are underlined.

Primer DNA sequence (5´🡪 3´) Restriction enzymes

Mutagenesis

narB_up-For AAATCTAGAGTCGATCCCGAACATGAGG XbaI

narB_up-Rev AAAGGATCCCGATCGGCAAGGAAACCAG BamHI

narB_down-For AAAGGATCCTAGAAGGCGACGGAATCAGG BamHI

narB_down-Rev AAAGAATTCCAGCCGAGTAAGGAGATGGAC EcoRI

narB_EXT-For CTCTTCGACATGGACGGCACCATC

narB_EXT-Rev ATTACGACCGCTTCGTCTTCTCC

narB_IN-For GCTGATAGATGACGGGATGACAC

narB_IN-Rev ATCCTGCTTGCCGCTCTGTTTCTC

Complementation

narB_compl-For AAATCTAGAAAGATGGAGACGCCGCATCCGAC XbaI

narB_compl-Rev AAAAAGCTTCGCGCTCGAAAGCCTGTTGATGG HindIII

narB_compl-IN1 AGTCTTCGACGGTTTCGTTGAAGG

narB_compl-IN2 AATGGCCGCTGCCTGGAAATGC

narB_compl-IN3 TCCGCTCGAGGAGATCGTTG
